# Supplementary material for: Development of AI-Based Predictive Models for Osteoporosis Diagnosis in Postmenopausal Women from Panoramic Radiographs
Source: J Clin Med. 2025 Jun 23;14(13):4462. doi: 10.3390/jcm14134462 (PMC12249935; doi:10.3390/jcm14134462)
Supplement: Supplementary file 1 [file jcm-14-04462-s001.zip › jcm-3608713-supplementary.pdf]

Supplementary Table S1: Exclusion Criteria

Criteria	Details
Systemic diseases	Diseases that severely affect bone metabolism (e.g., Cushing's syndrome, Addison's disease, type 1 diabetes mellitus, leukemia, pernicious anemia, malabsorption syndromes, chronic liver disease, rheumatoid arthritis)
Known infections	Known infection with HIV or viral hepatitis
Radiation therapy	History of local radiation therapy within the last five years
Limited mental or language capacity	Limited mental capacity or language skills impeding understanding of study information or consent
Severe medical or psychiatric conditions	Severe acute or chronic medical or psychiatric conditions or laboratory abnormalities that could interfere with trial participation or interpretation of results
Gender	Male

Supplementary Table S2: Inclusion Criteria

Criteria	Details
Age	Adults aged 50 years and older
Menopause	Self-reported menopause, defined as the permanent cessation of ovulation for at least one year
DXA examination	DXA examination at the hip and lumbar spine performed within the previous 12 months
OPT images	Availability of high-quality OPT images
Clinical diagnosis	Documented clinical diagnosis regarding bone health
Medical and dental records	Presence of comprehensive medical and dental records
Osteoporosis diagnosis	Confirmed diagnosis of osteoporosis based on DXA with a T-score of -2.5 or lower at the hip or spine
Healthy controls	T-score greater than or equal to -1 (no osteoporosis)
Temporal discrepancy	Temporal discrepancy between OPT and DXA analysis not longer than 4 months
Dichotomization	Individuals classified as osteoporotic (T-score $\leq$ -2.5) or non-osteoporotic (T-score $\geq$ -1)

Supplementary Table S3: Performance of Logistic Regression, SVC, Gaussian NB, and Decision Tree on test and external datasets using the classical radiomics approach, with AUC, accuracy, recall, and specificity reported for each model.

Model	Test AUC	Test Accuracy	Test Recall	Test Specificity	External AUC	External Accuracy	External Recall	External Specificity
**Logistic Regression**	0.5556 [0.5000, 0.6389]	0.5806 [0.4194, 0.7419]	1.0000 [1.0000, 1.0000]	0.0000 [0.0000, 0.0000]	0.5147 [0.5000, 0.5484]	0.6415 [0.5094, 0.7736]	1.0000 [1.0000, 1.0000]	0.0000 [0.0000, 0.0000]
**SVC**	0.4957 [0.3034, 0.6738]	0.4194 [0.2581, 0.5806]	0.0000 [0.0000, 0.0000]	1.0000 [1.0000, 1.0000]	0.3638 [0.2608, 0.4634]	0.3585 [0.2264, 0.4906]	0.0000 [0.0000, 0.0000]	1.0000 [1.0000, 1.0000]
**Gaussian NB**	0.5556 [0.5000, 0.6389]	0.5806 [0.4194, 0.7419]	1.0000 [1.0000, 1.0000]	0.0000 [0.0000, 0.0000]	0.5147 [0.5000, 0.5484]	0.6415 [0.5094, 0.7736]	1.0000 [1.0000, 1.0000]	0.0000 [0.0000, 0.0000]
**Decision Tree**	0.5812 [0.4138, 0.7501]	0.6129 [0.4516, 0.7742]	0.7778 [0.5713, 0.9444]	0.3846 [0.1111, 0.6667]	0.5000 [0.5000, 0.5000]	0.3585 [0.2264, 0.4906]	0.0000 [0.0000, 0.0000]	1.0000 [1.0000, 1.0000]

Supplementary Table S4 : This table summarizes the mean area under the ROC curve (AUC) along with the standard deviation (± SD) for each supervised machine learning classifier applied to deep radiomic features extracted using five different convolutional neural network (CNN) architectures: InceptionV3, ResNet50, VGG16, EfficientNet, and DenseNet. The results provide a comparative overview of the discriminative performance of each classifier-CNN combination, highlighting variability in model robustness across feature extraction backbones.

Model	InceptionV3	ResNet50	VGG16	EfficientNet	DenseNet
Logistic Regression	0.737 ± 0.109	0.747 ± 0.108	0.826 ± 0.068	0.821 ± 0.060	0.826 ± 0.059
Random Forest	0.692 ± 0.071	0.697 ± 0.071	0.761 ± 0.088	0.782 ± 0.107	0.788 ± 0.115
SVM	0.590 ± 0.089	0.576 ± 0.067	0.665 ± 0.220	0.688 ± 0.115	0.686 ± 0.114
Naive Bayes	0.541 ± 0.057	0.543 ± 0.063	0.647 ± 0.043	0.568 ± 0.053	0.584 ± 0.057
KNN	0.566 ± 0.108	0.521 ± 0.063	0.704 ± 0.071	0.664 ± 0.136	0.659 ± 0.139
Decision Tree	0.573 ± 0.143	0.583 ± 0.078	0.666 ± 0.070	0.613 ± 0.133	0.608 ± 0.131

**Gradient Boosting**	0.718 ± 0.133	0.715 ± 0.040	0.818 ± 0.114	0.734 ± 0.096	0.736 ± 0.086

Supplementary Table S5: Comparison of performance metrics between internal test set and external validation across different CNN architectures using logistic regression.

CNN Model	Mean AUC ± SD	AUC (Test)	AUC (External)	Accuracy (Test)	Accuracy (External)	Recall (Test)	Recall (External)	Specificity (Test)	Specificity (External)
InceptionV3	0.737 ± 0.109	0.580 [0.340, 0.814]	0.605 [0.454, 0.755]	0.600 [0.400, 0.800]	0.589 [0.464, 0.714]	0.867 [0.688, 1.000]	0.629 [0.469, 0.778]	0.200 [0.000, 0.500]	0.524 [0.300, 0.739]
ResNet 50	0.747 ± 0.108	0.687 [0.442, 0.904]	0.610 [0.457, 0.754]	0.640 [0.440, 0.840]	0.536 [0.410, 0.661]	0.867 [0.688, 1.000]	0.571 [0.412, 0.730]	0.300 [0.000, 0.583]	0.476 [0.261, 0.706]

VGG16	0.826 ± 0.068	0.647 [0.397, 0.864]	0.473 [0.302, 0.632]	0.640 [0.440, 0.800]	0.446 [0.321, 0.571]	0.667 [0.428, 0.895]	0.457 [0.294, 0.618]	0.600 [0.250, 0.889]	0.429 [0.211, 0.636]
Efficient Net	0.821 ± 0.060	0.747 [0.506, 0.922]	0.683 [0.538, 0.819]	0.680 [0.480, 0.840]	0.589 [0.464, 0.714]	0.733 [0.467, 0.938]	0.486 [0.322, 0.667]	0.600 [0.250, 0.900]	0.762 [0.550, 0.933]
DenseNet	0.826 ± 0.059	0.7667 [0.5383, 0.9333]	0.7224 [0.5735, 0.8516]	0.6800 [0.4800, 0.8400]	0.5714 [0.4464, 0.7143]	0.7333 [0.4667, 0.9375]	0.4286 [0.2647, 0.6000]	0.6000 [0.2500, 0.9000]	0.8095 [0.6087, 0.9565]

Supplementary Table S6: Model performance of the 5 CNN architectures using four types of transfer learning techniques in internal test

CNN Architecture	Accuracy	AUC	Sensitivity	Specificity	Transfer learning
**EfficientNet-b0**	0.440 (95% CI: 0.240-0.640)	0.468 (95% CI: 0.220-0.699)	0.000 (95% CI: 0.000-0.000)	1.000 (95% CI: 1.000-1.000)	Hybrid
**VGG16**	0.560 (95% CI: 0.360-0.760)	0.558 (95% CI: 0.331-0.795)	1.000 (95% CI: 1.000-1.000)	0.000 (95% CI: 0.000-0.000)	

**ResNet-50**	0.440 (95% CI: 0.240-0.640)	0.656 (95% CI: 0.417-0.853)	1.000 (95% CI: 1.000-1.000)	0.000 (95% CI: 0.000-0.000)	
**DenseNet-121**	0.520 (95% CI: 0.320-0.720)	0.604 (95% CI: 0.357-0.812)	0.714 (95% CI: 0.437-0.923)	0.273 (95% CI: 0.000-0.571)	
**InceptionV3**	0.880 (95% CI: 0.720-1.000)	0.825 (95% CI: 0.596-1.000)	1.000 (95% CI: 1.000-1.000	0.727 (95% CI: 0.437-1.000)	
**EfficientNet-b0**	0.600 (95% CI: 0.400-0.760)	0.539 (95% CI: 0.295-0.763)	1.000 (95% CI: 1.000-1.000)	0.091 (95% CI: 0.000-0.300)	Non-Hybrid
**VGG16**	0.560 (95% CI: 0.360-0.760)	0.584 (95% CI: 0.351-0.831)	1.000 (95% CI: 1.000-1.000)	0.000 (95% CI: 0.000-0.000)	
**ResNet-50**	0.600 (95% CI: 0.400-0.800)	0.708 (95% CI: 0.500-0.907)	0.929 (95% CI: 0.769-1.000)	0.182 (95% CI: 0.000-0.429)	
**DenseNet-121**	0.560 (95% CI: 0.360-0.760)	0.825 (95% CI: 0.647-0.974)	1.000 (95% CI: 1.000-1.000)	0.000 (95% CI: 0.000-0.000)	
**InceptionV3**	0.560 (95% CI: 0.360-0.760)	0.565 (95% CI: 0.327-0.799)	1.000 (95% CI: 1.000-1.000)	0.000 (95% CI: 0.000-0.000)	
**EfficientNet-b0**	0.600 (95% CI: 0.400-0.800)	0.773 (95% CI: 0.559-0.944)	0.786 (95% CI: 0.545-1.000)	0.364 (95% CI: 0.100-0.667)	50% Fine-Tuning
**VGG16**	0.640 (95% CI: 0.440-0.800)	0.649 (95% CI: 0.396-0.868)	1.000 (95% CI: 1.000-1.000)	0.182 (95% CI: 0.000-0.417))	
**ResNet-50**	0.720 (95% CI: 0.520-0.880)	0.883 (95% CI: 0.718-1.000)	0.929 (95% CI: 0.778-1.000)	0.455 (95% CI: 0.154-0.750)	
**DenseNet-121**	0.640 (95% CI: 0.440-0.800)	0.695 (95% CI: 0.471-0.891)	0.857 (95% CI: 0.625-1.000)	0.364 (95% CI: 0.100-0.636)	
**InceptionV3**	0.760 (95% CI: 0.600-0.920)	0.727 (95% CI: 0.494-0.938)	0.929 (95% CI: 0.769-1.000)	0.545 (95% CI: 0.250-0.833)	

**EfficientNet-b0**	0.600 (95% CI: 0.400-0.800)	0.773 (95% CI: 0.559-0.944)	0.786 (95% CI: 0.545-1.000)	0.364 (95% CI: 0.100-0.667)	Scratch Fine-Tuning
**VGG16**	0.560 (95% CI: 0.360-0.760)	0.500 (95% CI: 0.500-0.500)	1.000 (95% CI: 1.000-1.000)	0.000 (95% CI: 0.000-0.000)	
**ResNet-50**	0.440 (95% CI: 0.240-0.640)	0.545 (95% CI: 0.302-0.780)	0.000 (95% CI: 0.000-0.000)	1.000 (95% CI: 1.000-1.000)	
**DenseNet-121**	0.600 (95% CI: 0.400-0.800)	0.753 (95% CI: 0.545-0.931)	0.786 (95% CI: 0.556-1.000)	0.364 (95% CI: 0.091-0.667))	
**InceptionV3**	0.600 (95% CI: 0.400-0.760)	0.740 (95% CI: 0.522-0.929)	0.929 (95% CI: 0.769-1.000)	0.182 (95% CI: 0.000-0.444)	

Supplementary table 7 : Model performance of the 5 CNN architectures using four types of transfer learning techniques in external test

CNN Architecture	Accuracy	AUC	Sensitivity	Specificity	Transfer learning
**EfficientNet-b0**	0.375 (95% CI: 0.250-0.500)	0.437 (95% CI: 0.281-0.603)	0.000 (95% CI: 0.000-0.000)	1.000 (95% CI: 1.000-1.000)	Hybrid
**VGG16**	0.625 (95% CI: 0.500-0.750)	0.600 (95% CI: 0.439-0.745)	1.000 (95% CI: 1.000-1.000)	0.000 (95% CI: 0.000-0.000)	
**ResNet-50**	0.571 (95% CI: 0.446-0.696)	0.786 (95% CI: 0.654-0.894)	0.905 (95% CI: 0.769-1.000)	0.371 (95% CI: 0.212-0.517)	
**DenseNet-121**	0.607 (95% CI: 0.482-0.750)	0.566 (95% CI: 0.398-0.725)	0.886 (95% CI: 0.784-0.972)	0.143 (95% CI: 0.000-0.316)	

**InceptionV3**	0.554 (95% CI: 0.411-0.679)	0.618 (95% CI: 0.459-0.774)	0.514 (95% CI: 0.343-0.677)	0.619 (95% CI: 0.389-0.826)	
**EfficientNet-b0**	0.607 (95% CI: 0.482-0.732)	0.639 (95% CI: 0.487-0.783)	0.771 (95% CI: 0.629-0.900)	0.333 (95% CI: 0.143-0.539)	Non-Hybrid
**VGG16**	0.625 (95% CI: 0.500-0.750)	0.631 (95% CI: 0.474-0.781)	1.000 (95% CI: 1.000-1.000)	0.000 (95% CI: 0.000-0.000)	
**ResNet-50**	0.679 (95% CI: 0.554-0.804)	0.732 (95% CI: 0.585-0.860)	1.000 (95% CI: 1.000-1.000)	0.143 (95% CI: 0.000-0.316)	
**DenseNet-121**	0.625 (95% CI: 0.500-0.750)	0.727 (95% CI: 0.593-0.852)	1.000 (95% CI: 1.000-1.000)	0.000 (95% CI: 0.000-0.000)	
**InceptionV3**	0.607 (95% CI: 0.482-0.732)	0.668 (95% CI: 0.506-0.803)	0.971 (95% CI: 0.909-1.000)	0.000 (95% CI: 0.000-0.000)	
**EfficientNet-b0**	0.679 (95% CI: 0.554-0.786)	0.680 (95% CI: 0.518-0.820)	0.914 (95% CI: 0.816-1.000)	0.286 (95% CI: 0.105-0.476)	50% Fine-Tuning
**VGG16**	0.607 (95% CI: 0.482-0.732)	0.561 (95% CI: 0.403-0.716)	0.943 (95% CI: 0.854-1.000)	0.048 (95% CI: 0.000-0.150)	
**ResNet-50**	0.661 (95% CI: 0.536-0.786)	0.615 (95% CI: 0.463-0.759)	0.886 (95% CI: 0.765-0.973)	0.286 (95% CI: 0.095-0.480)	
**DenseNet-121**	0.554 (95% CI: 0.429-0.679)	0.567 (95% CI: 0.406-0.712)	0.714 (95% CI: 0.571-0.850)	0.286 (95% CI: 0.100-0.500)	
**InceptionV3**	0.661 (95% CI: 0.536-0.768)	0.652 (95% CI: 0.505-0.788)	0.829 (95% CI: 0.694-0.944)	0.381 (95% CI: 0.182-0.579)	
**EfficientNet-b0**	0.589 (95% CI: 0.464-0.714)	0.537 (95% CI: 0.377-0.690)	0.686 (95% CI: 0.529-0.839)	0.429 (95% CI: 0.238-0.637)	Scratch Fine-Tuning
**VGG16**	0.625 (95% CI: 0.500-0.750)	0.631 (95% CI: 0.474-0.781)	1.000 (95% CI: 1.000-1.000)	0.000 (95% CI: 0.000-0.000)	

**ResNet-50**	0.661 (95% CI: 0.536-0.786)	0.615 (95% CI: 0.463-0.759)	0.886 (95% CI: 0.765-0.973)	0.286 (95% CI: 0.095-0.480)	
**DenseNet-121**	0.554 (95% CI: 0.429-0.679)	0.567 (95% CI: 0.406-0.712)	0.714 (95% CI: 0.571-0.850)	0.286 (95% CI: 0.100-0.500)	
**InceptionV3**	0.607 (95% CI: 0.482-0.732)	0.668 (95% CI: 0.506-0.803)	0.971 (95% CI: 0.909-1.000)	0.000 (95% CI: 0.000-0.000)	
